# Strategies to Improve Luminescence Efficiency and Stability of Blue Perovskite Light‐Emitting Devices

**DOI:** 10.1002/smsc.202000048

**Published:** 2021-03-23

**Authors:** Xiao-Yan Qian, Ying-Yi Tang, Wei Zhou, Yang Shen, Ming-Lei Guo, Yan-Qing Li, Jian-Xin Tang

**Affiliations:** ^1^ School of Physics and Electronic Science Ministry of Education Nanophotonics & Advanced Instrument Engineering Research Center East China Normal University Shanghai 200062 China; ^2^ Jiangsu Key Laboratory for Carbon-Based Functional Materials & Devices Institute of Functional Nano & Soft Materials (FUNSOM) Soochow University Suzhou Jiangsu 215123 P. R. China; ^3^ Macao Institute of Materials Science and Engineering (MIMSE) Macau University of Science and Technology Taipa 999078 Macau China

**Keywords:** blue perovskite light-emitting diodes, external quantum efficiencies, luminescence, photoluminescence, stabilities

## Abstract

Metal halide perovskite materials hold great potential as a core element in full‐color display and lighting applications due to their unique optoelectronic properties, such as high photoluminescence efficiency, good color purity, tunable bandgap, and low‐temperature solution processability. However, the performance of blue perovskite light‐emitting diodes (PeLEDs) lags far behind their red and green counterparts, impeding their practical use. Herein, the recent progress on blue PeLEDs based on different restrictions and some feasible strategies to improve luminescence efficiency and stability are summarized. The methods to optimize blue‐emission perovskite materials with different dimensions are addressed. The major factors affecting luminescence stability, the approaches to balance charge injection and minimize the optical loss are also discussed. Several problems in blue PeLEDs, particularly the toxicity of lead‐based perovskites and the alternative solutions of lead‐free perovskites, are emphasized. Finally, the perspective of future development in blue PeLEDs is provided.

## Graphical abstract

The recent developments in blue perovskite light‐emitting diodes are reviewed in terms of promotion for luminescence efficiency and stability. The strategies including optimization for film quality and device structure with balanced charge injection, attenuation of toxicity, suppression of optical loss, and regulation of emission wavelength are emphasized.

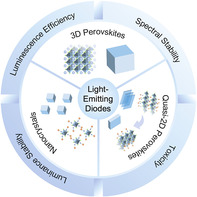

## 1 Introduction

In recent years, metal‐halide perovskite materials have been regarded as the promising candidate for the use in light‐emitting diodes (LEDs)^[^ ^1^, ^2^, ^3^, ^4^, ^5^, ^6^, ^7^, ^8^, ^9^ ^]^ due to their unique characteristics such as high photoluminescence quantum yield (PLQY), excellent color purity, tunable bandgap, and simple solution processability.^[^ ^10^, ^11^, ^12^, ^13^, ^14^, ^15^, ^16^ ^]^ According to the dimensions of perovskite materials, they can be categorized as 0D, 1D, 2D, and 3D, among which quasi‐2D perovskites are most widely used at present.^[^ ^17^, ^18^, ^19^, ^20^, ^21^, ^22^, ^23^ ^]^ For the potential applications in full‐color displays and lightings, perovskite LEDs (PeLEDs) not only overcome the shortcomings of inorganic LEDs that cannot be used for flexible displays,^[^ ^24^, ^25^, ^26^, ^27^ ^]^ but also show excellent color purity as compared with that of organic LEDs.^[^ ^28^ ^]^ Since the first report of red and green PeLEDs at room temperature in 2014 by Tan et al.,^[^ ^29^ ^]^ rapid progress in external quantum efficiencies (EQEs) has been made. PeLEDs with green, red and near‐infrared emissions have gained tremendous developments with EQEs exceeding 20%.^[^ ^30^, ^31^, ^32^ ^]^ However, the performance of blue PeLEDs still lags far behind the other counterparts in the aspects of electroluminescent (EL) efficiency and spectral stability.^[^ ^33^, ^34^, ^35^ ^]^ Moreover, blue PeLEDs suffer from the poor lifetime as compared with that of traditional inorganic, organic, and quantum dot LEDs. Therefore, the effective schemes toward high efficiency and long lifetime of blue PeLEDs are highly desirable to promote their commercialization in the new‐generation full‐color displays.^[^ ^36^ ^]^

**Figure 1:**
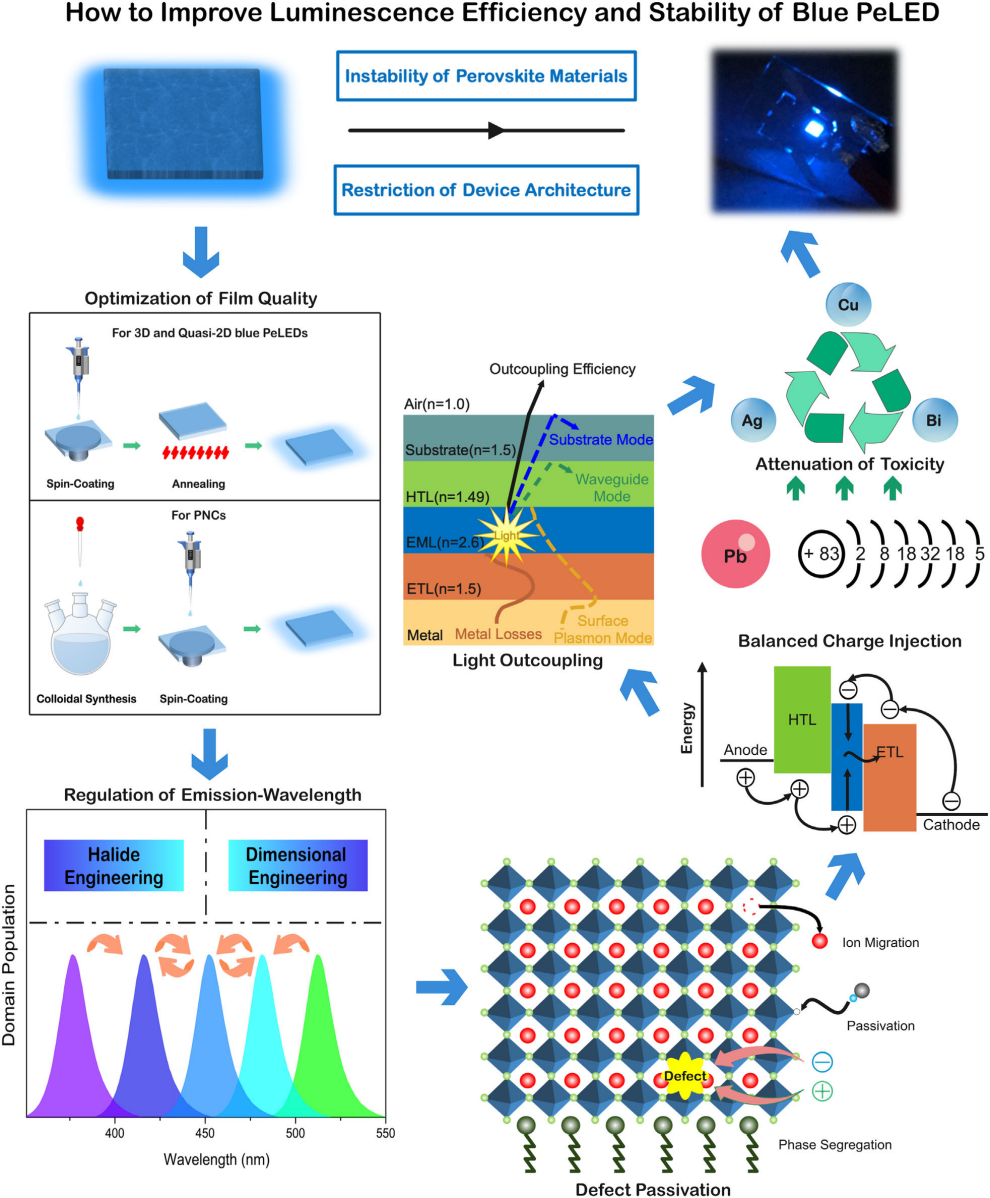
Schematic illustration of the strategies to improve luminescence efficiency and stability of blue PeLED.

In this Review, we focus on the strategies that improve the EL efficiency and stability of blue PeLEDs. To be specific, the proposed strategies are summarized based on the problems in blue PeLEDs and the corresponding solutions (**Figure** **1**). We first begin with a summary of the methods to optimize blue‐emission perovskite materials based on their crystal dimension, including 3D, quasi‐2D, and nanocrystals (NCs). Then, we present the factors that affect the spectral stability and luminance stability (or lifetime) based on different degradation mechanisms. The origins of unbalanced charge injection and optical loss are discussed, and the possible solutions to charge transport and light outcoupling are provided. The current progress made on eliminating the toxicity problem of Pb‐based perovskites is also analyzed along with the alternative solution of Pb‐free perovskites. Finally, the perspectives for future development in blue PeLEDs are provided.

## 2 Status Quo of Blue Perovskite Materials

### 2.1 3D/Quasi‐2D Perovskites

**Figure 2:**
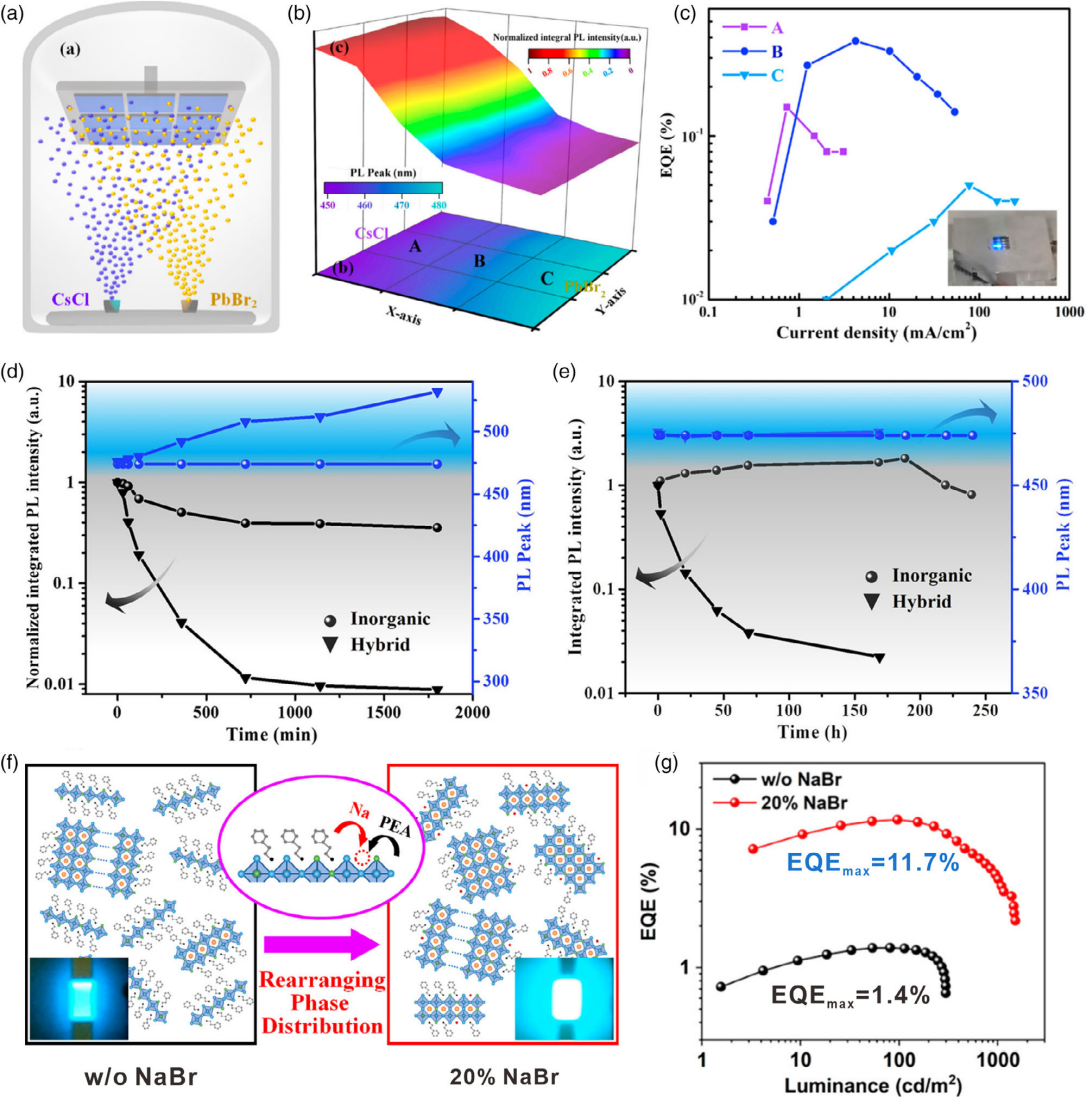
Fabrication of all‐inorganic CsPbBr_*x*_Cl_3−*x*_ perovskite films. a) Schematic illustration of dual‐source coevaporation of CsCl and PbBr_2_. b) Spatially resolved PL peak and intensity of the coevaporated CsPbBr_*x*_Cl_3−*x*_ perovskite films. c) EQE versus current density for PeLEDs based on samples A, B, and C as indicated in (b). d) Thermal stability of all‐inorganic and hybrid perovskites under the aging conditions of 100 °C continuous heating in N_2_. e) Ultraviolet (UV) irradiation stability under 10 mW cm^−2^, 365 nm ultraviolet lamp irradiation in N_2_. a–e) Reproduced with permission.^[^ ^40^ ^]^ Copyright 2019, American Chemical Society. f) Schematic illustration of the phase rearrangement by adding Na^+^ in quasi‐2D perovskites. g) EQE versus luminance of PeLEDs without and with 20% NaBr. f,g) Reproduced with permission.^[^ ^45^ ^]^ Copyright 2020, American Chemical Society.

Metal halide perovskites have a typical chemical formula of ABX_3_, where A is a monovalent organic or inorganic cation (e.g., methylammonium [MA^+^], formamidinium [FA^+^], or Cs^+^), and B is a bivalent metal cation (e.g., Pb^2+^ or Sn^2+^), and X is a halide anion (Cl^−^, Br^−^, or I^−^). Usually, a mixture of different ions (e.g., mixed MA^+^, FA^+^, and Cs^+^, mixed Pb^2+^ and Sn^2+^, and mixed halide) is used to form each site in the perovskite crystals, leading to a large tunability of electronic and optical properties.^[^ ^37^ ^]^ 3D perovskites possess superior advantages of long carrier diffusion length, excellent charge mobility, low trap states, and so on. Increasing the chlorine content in chloride/bromide (Cl/Br)‐mixed perovskites can tune the emission wavelength toward blue‐light region (**Table** **1**). Since 2015, as shown in Table 1, the indium tin oxide (ITO) is usually used as the transparent anode, a layer of zinc oxide nanoparticles (ZnO‐NPs) is used as an electron‐transport layer, and poly (2,3‐dihydrothieno‐1,4‐dioxin):poly styrenesulfonate (PEDOT:PSS), *N*,*N*′‐bis(4‐butylphenyl)‐*N*,*N*‐bis(phenyl)‐benzi (TPD), and *N*,*N*‐bis(1‐naphthyl)‐*N*,*N*‐diphenylbenzidine (NPD), poly[bis(4‐phenyl)(2,4,6‐trimethylphenyl)amine (PTAA) are used as a hole‐transport layer, respectively. Kumawat et al. used the MAPbBr_1.08_Cl_1.92_‐based 3D perovskite as an emitter to achieve the first blue PeLED with an EL peak (*λ*_EL_) at 482 nm.^[^ ^16^ ^]^ However, this device exhibits a low EQE of only 0.0003% due to inadequate film coverage and high defect density of the Cl/Br‐mixed perovskites. To solve the problem of low efficiency in blue PeLEDs, Kim et al. synthesized the Cs_10_(MA_0.17_FA_0.83_)_100 − *x*_PbBr_1.5_Cl_1.5_ perovskite by doping Cs^+^ cations into the MA/FA‐based perovskite, improving the surface coverage of traditional 3D perovskite films and reducing the surface defects.^[^ ^38^ ^]^ A blue PeLED (*λ*_EL_ = 475 nm) was obtained with a maximum luminance (*L*_max_) of 3567 cd m^−2^ and a maximum EQE (EQE_max_) of 1.7%. However, the phase separation and ion migration behaviors were observed in this halogen‐mixed 3D perovskite, impeding the luminescence stability. The luminance increased to 948.5 cd m^−2^ during the first 25 min and then decreased rapidly to 88.5 cd m^−2^ after 150 min operation. To improve the operational stability of blue PeLEDs, Yuan et al. developed a cocktail strategy that incorporated multiple cations Rb/FA/phenethylamine (PEA)/K into the inorganic CsPb(Cl/Br)_3_ perovskite, and the synergetic effect of multiple cations on the ion‐induced crystallization ameliorated the film quality with the improved radiative recombination and operational stability.^[^ ^39^ ^]^ When further using an insulator/perovskite/insulator structure to reduce hole‐caused leakage current and inhibit exciton quenching, blue PeLEDs (*λ*_EL_ = 484 nm) achieved a *L*_max_ of 4015 cd m^−2^, an EQE_max_ of 2.01%, and a half‐lifetime (*T*_50_) of >300 min under continuous operation conditions. However, the organic components contained in mixed perovskites inevitably impose the intrinsic drawbacks in thermal stability of perovskite materials and spectral stability of blue‐light emission. Du et al. successfully prepared all‐inorganic CsPb(Br/Cl)_3_ perovskite films with the emission wavelengths ranging from 450 to 480 nm using the dual‐source coevaporation of CsCl and PbBr_2_ (**Figure** **2**a,b).^[^ ^40^ ^]^ The vacuum deposition method can avoid the disadvantages of the limited solubility of cesium salt in the solution method, and the Cl/Br ratio can be continuously adjusted to precisely achieve the standard blue emission. Moreover, the thermally evaporated perovskite film exhibited good film morphology, and an EQE_max_ of 0.38% was realized for deep blue PeLEDs (*λ*_EL_ = 468 nm) (Figure 2c–e).

**Table 1:** Summary of blue PeLEDs based on 3D and quasi‐2D perovskites

Perovskite material	Device structure	EL [nm]	EQE_max_ [%]	*L*_max_ [cd m^−2^]	Ref.
MAPbBr_1.08_Cl_1.92_	ITO/PEDOT:PSS/3D perovskite/PC_61_PbM/Ag	482	0.0003	1.72	[16]
Cs_10_(MA_0.17_FA_0.83_)_100−*x*_PbBr_1.5_Cl_1.5_	ITO/ZnO‐NPs/3D perovskite /α‐NPD/MoO_3_/Al	475	1.7	3567	[38]
(Cs/Rb/FA/PEA/K)Pb(Cl/Br)_3_	ITO/LiF/3D perovskite/LiF/Bphen/LiF/Al	484	2.01	4015	[39]
CsPbBr_*x*_Cl_3 − *x*_	ITO/NiO_*x*_/3D perovskite/TPBi/LiF/Al	468	0.38	121	[40]
(BA)_2_(MA)_2_Pb_3_Br_7_Cl_3_	ITO/PEDOT:PSS/poly‐TPD/quasi‐2D/TPBi/LiF/Al	468	0.01	21	[42]
PA_2_(CsPbBr_3_)_*n*−1_PbBr_4_	ITO/PEDOT:PSS/quasi‐2D/TmPyPB/Cs_2_CO_3_/Al	505	3.6	7320	[44]
–	ITO/NiO_*x*_/PTAA/PVK/quasi‐2D/TPBi/LiF/Al	488	11.7	–	[45]

Despite the use of conventional 3D perovskites for blue emission, quasi‐2D perovskites have attracted tremendous attention in recent years due to their high chemical and light stability as well as large exciton binding energy caused by quantum confinement for the promoted radiative recombination efficiency (Table 1).^[^ ^41^, ^42^, ^43^, ^44^, ^45^ ^]^ By controlling the number (*n*) of corner‐sharing octahedra (PbI_6_) layers, Yuan et al. reported multilayered quasi‐2D PeLED operating at near‐infrared wavelengths with an EQE_max_ of 8.8%.^[^ ^41^ ^]^ In 2018, Hu et al. fabricated an efficient deep‐blue PeLED (*λ*_EL_ = 468 nm) with a quasi‐2D perovskite of (BA)_2_(MA)_2_Pb_3_Br_7_Cl_3_, yielding an EQE_max_ of 0.01% and a *L*_max_ of 21 cd m^−2^.^[^ ^42^ ^]^ The variation of the *n* number in quasi‐2D perovskites can not only tune the energy transfer (ET) process, but also another process of charge transfer (CT). Shang et al. engineered the (PEA)_2_(MA)_*n*−1_Pb_*n*_I_3*n*+1_ Ruddlesden–Popper perovskite structure, demonstrating the effective CT in low pinhole‐density perovskite films.^[^ ^43^ ^]^ Alternatively, Chen et al. successfully used PA_2_(CsPbBr_3_)_*n*−1_PbBr_4_ to fabricate blue‐green PeLEDs (*λ*_EL_ = 505 nm) with an EQE_max_ of 3.6% and a *L*_max_ to 7320 cd m^−2^, which was attributed to the efficient CT by adopting an electron transport material of TmPyPB.^[^ ^44^ ^]^ In 2020, Pang et al. rearranged the low‐dimensional phase distribution by incorporating sodium ions (Na^+^) to partially substitute the large organic molecules in the Cl/Br‐mixed quasi‐2D perovskites (Figure 2f), which induced the suppression of the formation of *n* = 1 phase but the significant elevation of other small‐*n* phases (i.e., *n* = 2, 3, and 4).^[^ ^45^ ^]^ This rearrangement of phase distributions gave rise to effective radiative recombination and exciton ET in the quasi‐2D perovskite films, achieving the sky‐blue PeLED (*λ*_EL_ = 488 nm) with an EQE_max_ of 11.7% (Figure 2g).

Quasi‐2D PeLEDs usually exhibit multiple EL peaks and unstable colors due to the different ET efficiencies inside perovskite materials. To solve this problem, the anion mixing and quantum confinement engineering have been proposed. However, the EL spectra show the instability with the variation of the driving bias, which was caused by phase separation or the coexistence of multiple crystal domains. To further boost the spectral stability of quasi‐2D perovskite‐based blue PeLEDs, Xing et al. partially replaced the long ligand PEA with the short ligand IPA for reducing the van der Waals force between bulk organic cations and suppressing the formation of *n* = 1 phase, which was accompanied with the strengthened phase (*n* = 2, 3, 4) monodispersity.^[^ ^35^ ^]^ The optimized PeLED yielded a single emission peak (*λ*_EL_ = 490 nm), an average EQE of 1.0%, and a *L*_max_ of 2480 cd m^−2^.

### 2.2 Perovskite NCs

Perovskite NCs have also attracted considerable research interests due to their unique properties such as low‐temperature synthesis, controllable crystal size, and high dupability.^[^ ^46^ ^]^ An amount of colloidal synthesis strategies have been developed for perovskite NCs in recent years, including template‐assisted synthesis, ligand‐assisted precipitation (LARP), hot‐injection method, anion exchange reaction, microwave‐assisted synthesis, and so on.^[^ ^47^, ^48^, ^49^, ^50^, ^51^, ^52^, ^53^, ^54^, ^55^, ^56^, ^57^ ^]^ The performance of blue‐emission perovskite NCs mentioned in this section are shown in **Table** **2**.

**Table 2:** Summary of blue‐emission perovskite NCs with various synthesis methods

Synthesis method	Perovskite	NC size [nm]	PLQY [%]	Lifetime [ns]	FWHM [nm]	Ref.
Template‐assisted synthesis	CH_3_NH_3_PbBr_3_	6	20	–	–	[47]
Template‐assisted synthesis	CsPbBr_3_	6	70	9.8	25	[51]
LARP	CH_3_NH_3_PbX_3_	3.3	20	6.6	≈20	[52]
LARP	MAPbX_3_	5	49	4.83	≈20	[53]
Hot‐injection method	CsPbX_3_	4–15	50−90	1–29	50	[54]
Anion exchange reaction	CsPbX_3_	8.5	24	–	26	[49]
Anion exchange reaction	CsPbX_3_	4–15	20–80	–	10	[50]
Anion exchange reaction	CsPbBr_*x*_Cl_3 − *x*_	8	81	–	18	[55]
Microwave‐assisted synthesis	CsPbX_3_	15.1−23.7	90	1.9−135.7	14	[56]
Microwave‐assisted synthesis	CsPbX_3_	15.58	92	27	26	[57]

#### 2.2.1 Template‐Assisted Synthesis

**Figure 3:**
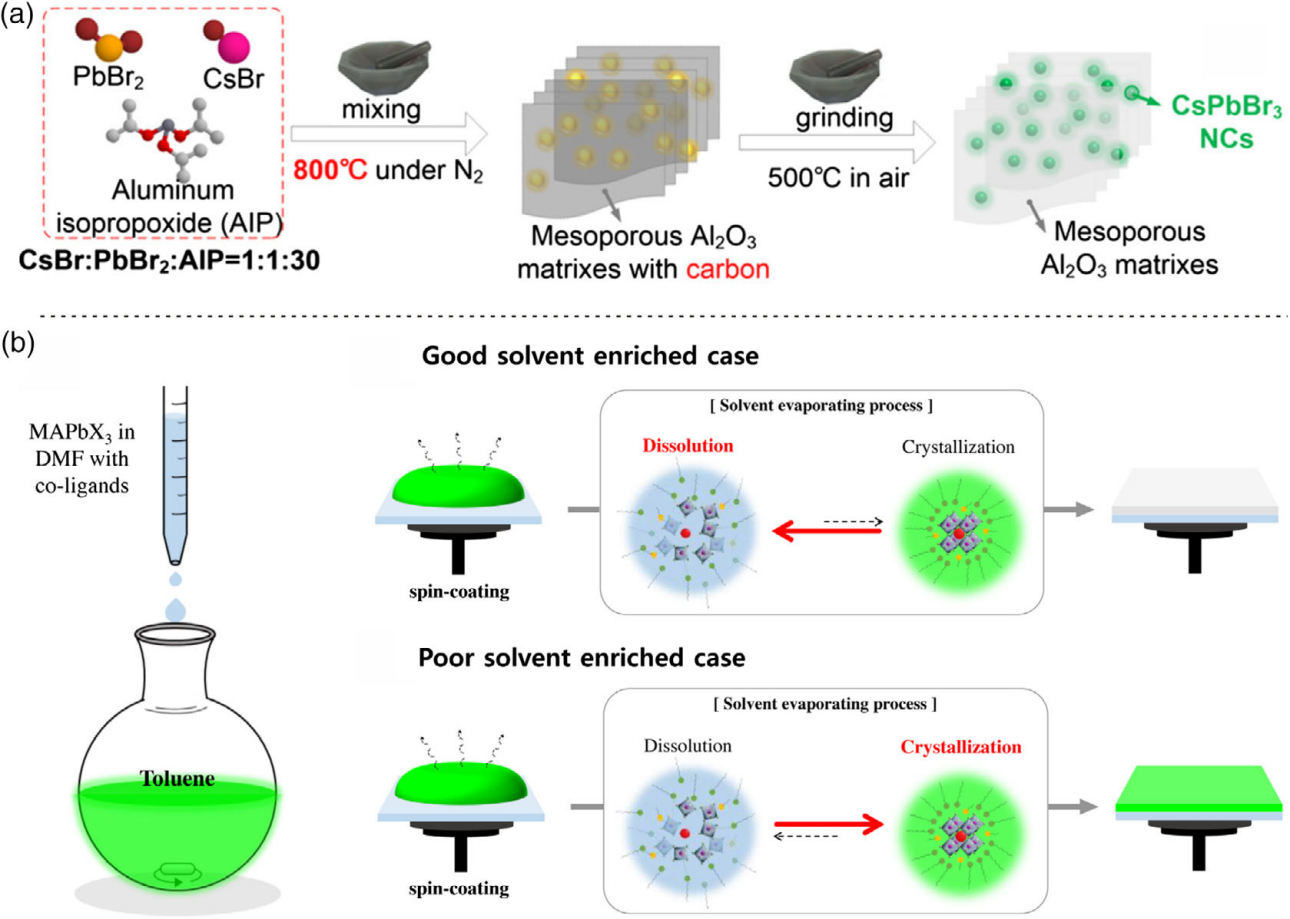
Synthesis of luminescent perovskite NCs. a) Schematic of high‐temperature solid‐state reaction process. Reproduced with permission.^[^ ^51^ ^]^ Copyright 2019, American Chemical Society. b) Schematic of postprocessable MAPbX_3_ NC solution by modified LARP method and two NC‐based films prepared by spin‐coating process. Reproduced with permission.^[^ ^53^ ^]^ Copyright 2020, Elesvier.

In 2014, Schmidt et al. used the template‐assisted method to synthesize luminous 6 nm‐sized MAPbBr_3_ perovskite NCs by choosing thin Al_2_O_3_ film as a mesoporous medium, which could be maintained stable in the solid state with a high quantum yield of 20%.^[^ ^47^ ^]^ However, many toxic organic solvents were consumed in the process of synthetic and purification processes, delivering the risk to human health and environmental pollution. Correspondingly, Wang et al. used the in situ formed mesoporous Al_2_O_3_ as a template to prepare highly emissive CsPbBr_3_ perovskite NCs through a solvent‐free high‐temperature solid‐state template confined growth strategy (**Figure** **3**a).^[^ ^51^ ^]^ The obtained CsPbBr_3_−Al_2_O_3_ powders possessed the PLQY up to 70%, narrow emission peak with a full width at half maximum (FWHM) of 25 nm, and outstanding thermal stability. This versatile method can be used for the synthesis of a large amount of perovskite NCs, and holds great potential for other CsPbX_3_ NCs by controlling the ratio of the halide ions.

#### 2.2.2 Ligand‐Assisted Precipitation

Due to the advantages of convenient operational temperatures, receptive equipment requirements, and ease of extensibility, the LARP method for synthesis of perovskite NCs is an affordable and potential pathway. It was found that perovskite NCs could exist stably in nonpolar organic solvents with the help of amine ions, whereas their optical properties were significantly influenced by several parameters, such as the ratios of precursor chemicals, temperature, capping agents, and solvent system of the precipitation process. On this basis, Zhang et al. successively proposed the LARP method which could synthesize bright MAPbX_3_ NCs directly.^[^ ^52^ ^]^ However, the perovskite NCs were synthesized by dropping the clear perovskite solution containing co‐ligands in the nonpolar aprotic solvents. The resulting solution still contained the solvents that could dissolve perovskites, such as *N*,*N*‐dimethylformamide (DMF), dimethylsulfoxide (DMSO), and ligands. To overcome this problem, Park et al. developed a modified LARP method that used DMF as a good solvent and *n*‐hexylamine (HxA) as a co‐ligand to synthesize organic–inorganic hybrid perovskite NCs (Figure 3b).^[^ ^53^ ^]^ The remained DMF would dissolve the MAPbX_3_ NC‐solid‐films if the amount of DMF could not reduce as much as possible. By controlling the ratio of DMF, the solid perovskite NC film were formed under the drying process.

#### 2.2.3 Hot‐Injection Method

Compared with other colloidal synthesis methods, the hot‐injection method provides a versatile way to prepare high‐luminescence colloidal perovskite NCs with tunable size, shape, and surface passivation. Protesescu et al. injected Cs‐oleic acid quickly into a noncoordinating solvent containing lead halide and ligand under the protection of N_2_ via the hot‐injection method.^[^ ^54^ ^]^ By changing the halide composition, the particle size, bandgap energy, and emission spectra of these perovskite NCs can be adjusted in the entire visible spectrum of 400–780 nm, implying a good solution to realize blue‐emission perovskite NCs.

#### 2.2.4 Anion Exchange Reaction

Postsynthetic halide exchange reactions in perovskite NCs, including cation exchange and anion exchange reaction, were regarded as a promising tool to manipulate the composition of perovskite NCs. In recent years, plentiful attempts have been carried out to exchange Cs^+^ cations or Pb^2+^ cations in CsPbX_3_ (X = Cl, Br, I) NCs, where the anion exchange reaction led to a blue shift for the routes of I^−^ → Br^−^ and Br^−^ → Cl^−^. Unfortunately, this method usually formed a new halide along with the decomposition of CsPbX_3_ NCs. In 2015, Akkerman et al. tuned the optical properties of CsPbX_3_ NCs via anion exchange reaction method with the preservation of the pristine crystal structure.^[^ ^49^ ^]^ Similarly, Nedelcu et al. presented a one‐step synthesis method of CsPbX_3_ NCs to form monodisperse colloidal nanotubes via anion exchange reaction, demonstrating the spectrally narrow and bright photoluminescence (PL) over the whole visible region.^[^ ^50^ ^]^ In 2020, Ye et al. used tetrabutylammonium *p*‐toluenesulfonate (TBSA) to trigger anion exchange reaction (Br^−^ → Cl^−^) due to the anion and cation synergistic effects of benzenesulfonates, leading to a blue shift from 456 to 409 nm.^[^ ^55^ ^]^ The resulting mixed‐halide CsPbBr_*x*_Cl_3−*x*_ blue PeLEDs achieved an EQE_max_ of 2.6%.

#### 2.2.5 Microwave‐Assisted Synthesis

The microwave‐assisted synthesis method can facilitate formation of the uniformly distributed cube‐shaped CsPbX_3_ NCs because of the homogeneous heating and rapid temperature growth, making it promising for achieving high‐quality perovskite NCs. In 2017, Liu et al. prepared all‐inorganic CsPbX_3_ NCs with high PLQY over 90% by optimizing the microwave‐assisted synthesis conditions, gaining blue PeLEDs with an EQE_max_ of 0.09% and color coordinate of (0.15,0.26).^[^ ^56^ ^]^ In 2020, Thesika and Murugan used microwave‐solvothermal synthesis method to improve the heterogeneous reaction at the interfaces for the formation of all‐inorganic CsPbX_3_ NCs with PLQY of 92% and narrow emission line widths.^[^ ^57^ ^]^ One unique feature of this method is that the synthesis time is dramatically reduced within 6 min as compared with traditional hot‐injection method that requires more than 3 h.

## 3 Challenges and Strategies for Blue PeLEDs

Despite rapid progress made in device efficiency, blue PeLEDs are still suffering from the inferior performance, including undesirable stability of color and luminance, imbalanced charge injection, inevitable optical loss, and toxicity. To date, the development of efficient and stable blue PeLEDs is urgently desired to be given high priority. The performances and device architectures of representative blue PeLEDs mentioned in this section are shown in **Table** **3**.

**Table 3:** Summary of materials engineering for efficient and stable blue PeLEDs

Structure	*λ*_EL_ [nm]	EQE_max_ [%]	*L*_max_ [cd m^−2^]	*T*_50_ [min]	Ref.
ITO/PEDOT:PSS/TFB/PFI/CsPbBr_3_:PEACl:YCl_3_/TPBi/LiF/Al	485	11.0	9040	≈80	[19]
	477	4.8	–	–	[19]
ITO/PEDOT:PSS/PEA_2_(Cs_1−*x*_EA_*x*_PbBr_3_)_2_PbBr_4_/TPBi/LiF/Al	488	12.1	2191	–	[58]
ITO/PVK/PFI/P‐PDABr_2_/PEABr/3TPYMB/Liq/Al	465	2.6	211	13.5	[59]
ITO/PEDOT:PSS/(Cs/FA/p‐F‐PEA)Pb(Cl/Br)_3_ /TPBi/LiF/Al	469	4.14	451	14	[60]
ITO/ PEDOT:PSS/TPD/BI_2_PbBr_4_/TPBi/LiF/Al	445	3.08	1315	210	[61]
ITO/NiO_*x*_/PEA_2−*x*_PA_2_(CsPbBr_3_)_*n*−1_PbBr_4_/TPBi/LiF/Al	488	7.51	1765	66	[68]
ITO/PEDOT:PSS/TFB/PFI/CsMn_*y*_Pb_1−*y*_Br_*x*_Cl_3−*x*_/TPBi/LiF/Al	466	2.12	245	–	[74]
ITO/PEDOT:PSS/PTAA/CsPbBr_3_(NCs)/MoO_*x*_/Ag	–	12.3	–	20	[76]
ITO/NiO_*x*_/PSSNa/PA_2_(CsPbBr_3_)_*n* − 1_PbBr_4_/TPBi/LiF/Al	492	1.45	4359	220	[77]
ITO/NiO/TFB/PVK/(FABr/CsBr/PbBr_2_)/TPBi/LiF/Al	483	9.5	54	250	[79]
ITO/p‐NiO/Cs_3_Cu_2_I_5_(NCs)/TPBi/LiF/Al	445	1.12	263.2	6480	[78]

### 3.1 Structural Modification for Efficient Emission

**Figure 4:**
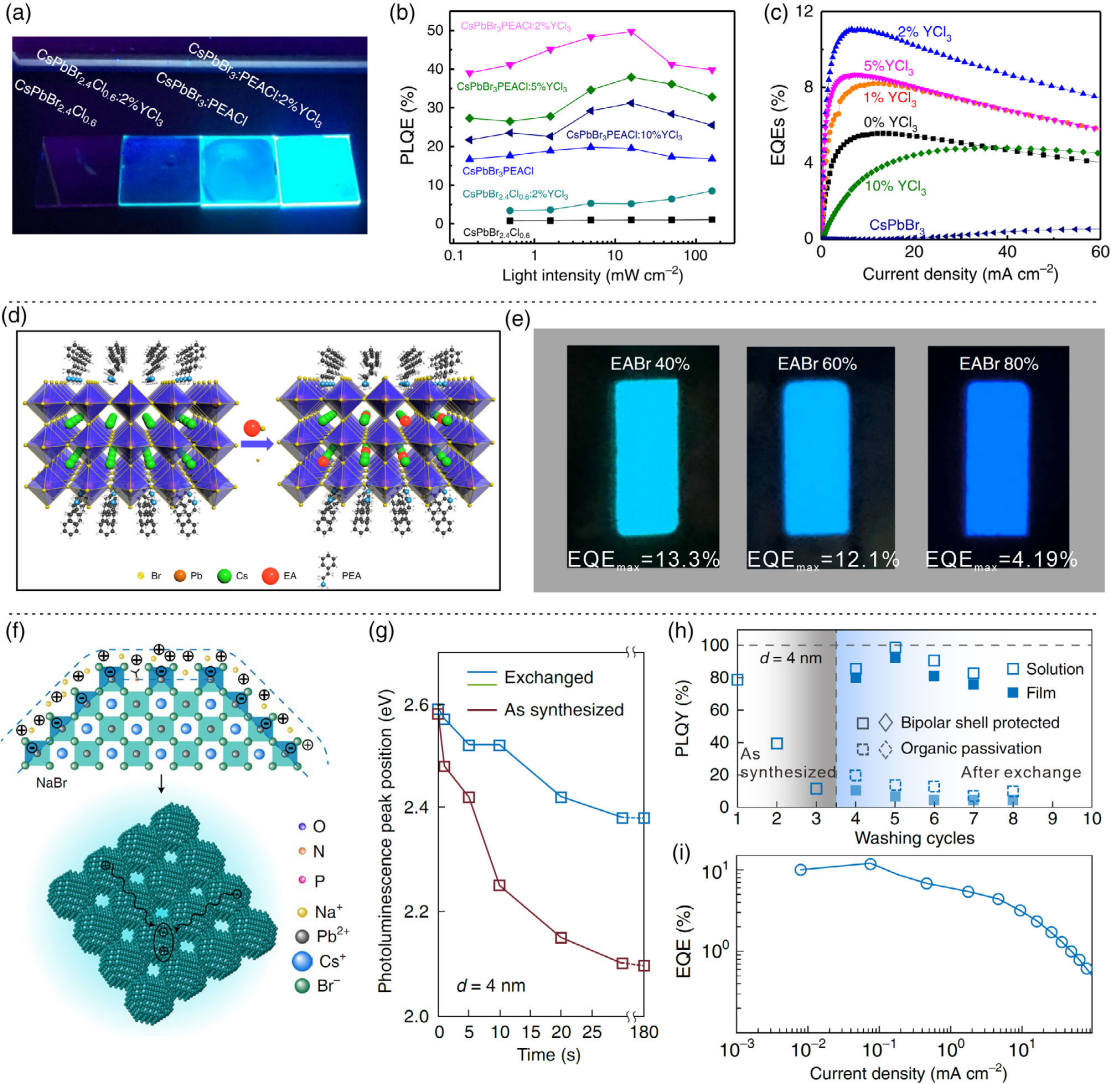
Cation incorporation in the blue‐emission perovskites. a) A photograph of the YCl_3_‐incorporated perovskite films under UV lamp. b) PLQYs versus light intensity of the perovskite films with different YCl_3_ compositions. c) EQE versus current density of sky‐blue PeLEDs with YCl_3_. a–c) Reproduced under the terms of the CC‐BY 4.0 license.^[^ ^19^ ^]^ Copyright 2019, The Authors, published by Springer Nature. d) Schematic of the EA cation doping in quasi‐2D perovskite. e) Device performance of PeLEDs with different EABr ratios. d,e) Reproduced under the terms of the CC‐BY 4.0 license.^[^ ^58^ ^]^ Copyright 2020, The Authors, published by Springer Nature. f) Schematic of bipolar‐shell resurfacing of neutral and sterically stabilized perovskite NCs. g) PL peak shift of bipolar‐shell‐stabilized CsPbBr_3_ perovskite NCs for monitoring the anion exchange rate at different reaction times. h) PLQYs of bipolar‐shell‐stabilized perovskite NCs and NC solid films. i) EQE of blue PeLED based on perovskite NC solid film. f–i) Reproduced with permission.^[^ ^76^ ^]^ Copyright 2020, Springer Nature.

To solve the bottleneck of the inferior charge transport, Wang et al. recently introduced PEACl (C_6_H_5_C_2_H_4_NH_3_Cl) and 2% YCl_3_ into 3D CsPbBr_3_ perovskite films (**Figure** **4**a,b) to improve the PLQY of the layered perovskite from 1.1% to 49.7% and to enhance the operational stability of perovskites on account of the suppression of ion migration. A sky‐blue PeLED (*λ*_EL_ = 485 nm) with an EQE_max_ of 11.0% and a blue PeLED (*λ*_EL_ = 477 nm) with an EQE_max_ of 4.8% were achieved by changing the content of YCl_3_ in the perovskite films (Figure 4c).^[^ ^19^ ^]^ Moreover, the sky‐blue PeLEDs with the incorporation of YCl_3_ exhibited the highly enhanced spectrum and luminance stabilities compared with 3D perovskite materials, which were ascribed to the dramatically suppressed ion migration and phase segregation in these mixed‐halide perovskite films under electric field. Soon afterward, Chu et al. refreshed the EQE values of sky‐blue PeLEDs by importing a large cation of CH_3_CH_2_NH^2+^ (EA^+^) to PEA_2_(CsPbBr_3_)_2_PbBr_4_ perovskite (Figure 4d), which inhibited the orbit coupling of Pb—Br and enlarged the optical bandgap for the emission changing from green (508 nm) into blue (466 nm).^[^ ^58^ ^]^ By optimizing the replacement of Cs^+^ cations with larger EA^+^ cations, a sky‐blue PeLED (*λ*_EL_ = 488 nm) with a high EQE_max_ of 12.1% was obtained, exhibiting excellent stability under light soaking and heating (Figure 4e).

Although remarkable development has been gained for sky‐blue PeLEDs with EQE over 10%, efficient and stable deep‐blue devices are rarely reported. A common way to fabricate deep‐blue PeLEDs is to reduce dimensional domain by adding larger organic ammonium (spacer). Yuan et al. used P‐PDAPbBr_4_ to ameliorate the poor coordination between traditional spacer (PEABr) and inorganic octahedron, leading to the optimized pure‐bromide PeLED with a deep‐blue emission (*λ*_EL_ = 465 nm) and an EQE_max_ of 2.6%.^[^ ^59^ ^]^ However, the introduction of larger organic ammonium would be adverse to the electrical conductivity of perovskite films. To avoid the influence of the incorporated organic additives on the transport properties of perovskite films, Shen et al. modified the substrate with potassium (K^+^) cations to improve radiative recombination and hole‐transport capabilities, arising from strong dipole interaction between K^+^ and halide ions.^[^ ^60^ ^]^ The K^+^‐triggered nucleation and grain growth promoted the formation of well‐packed (Cs/FA/p‐F‐PEA) Pb(Cl/Br)_3_ perovskite film with a PLQY of 34.8%, and thereby a deep‐blue PeLEDs (*λ*_EL_ = 469 nm) was obtained with an EQE_max_ of 4.14%. Yan et al. synthesized a stable pure layered (benzimidazole)_2_PbBr_4_ (BI_2_PbBr_4_) perovskite via the strong π–π interaction between benzene rings of the BI ligand, leading to the 2D‐layered perovskite film with a PLQY of 80% and the deep‐blue PeLED (*λ*_EL_ = 445 nm) with an EQE_max_ of 3.08% and a *L*_max_ of 1315 cd m^−2^.^[^ ^61^ ^]^

### 3.2 Luminescence Stability

Regardless of the significant boost in device efficiency, the luminescence stability of blue PeLEDs is still a considerable challenge for the practical use in full‐color display and lightings. Particularly, perovskite films incline to degrade under some special circumstances, such as light illumination, moisture exposure, heat and electrical bias, and the degradation pathways arise from various physical and chemical factors. Here, the origins for the luminescence instability and the key challenges are discussed in terms of composition engineering and surface passivation.^[^ ^50^, ^62^, ^63^, ^64^, ^65^, ^66^, ^67^, ^68^, ^69^, ^70^, ^71^, ^72^, ^73^, ^74^, ^75^, ^76^ ^]^

#### 3.2.1 Composition Engineering

It has been known that most defects occurring during the growth of perovskites are shallow defects, which do not contribute to carrier trapping and nonradiative recombination. As a result, perovskite materials have a high defect tolerance that enables huge freedom in the composition engineering with a variety of component choices to intentionally tune the electronic and optical properties. To improve the luminescence stability of perovskites, A‐site and B‐site doping in the ABX_3_ formula have been attempted. Swarnkar et al. presented a new strategy that used other metal ions as a substitute to replace Pb^2+^ (B‐site cation) for the phase stabilization of CsPbX_3_ perovskites, achieving stable optoelectronic devices with stable emission.^[^ ^69^ ^]^ Benefiting from high diffusion rate of halides, postsynthetic halide exchange reactions at room temperature have been widely used for the growth of perovskites.^[^ ^70^, ^71^, ^72^, ^73^ ^]^ However, the postsynthetic exchange of Pb^2+^ ions is not a simple and direct way. Mondal et al. prepared blue‐violet‐emitting (*λ* = 406 nm) CsPbCl_3_ perovskite NCs by treating CsPbCl_3_ with CdCl_2_ at room temperature, resulting in the easy replacement of Pb^2+^ ions with Cd^2+^ ions.^[^ ^73^ ^]^ The nonradiative carrier trapping centers were suppressed to facilitate radiative recombination process, and the Cd‐doped CsPbCl_3_ NCs exhibited near‐unity PLQY, high air stability, and high photostability. Meanwhile, Mn‐doped perovskite NCs have been proved to be highly efficient emitters, arising from the quick ET. Hou et al. added a small amount of Mn dopant into the perovskite NCs, which gave rise to the three‐fold increase in PLQY and maintained robust deep‐blue emission (*λ*_EL_ = 466 nm) with an EQE_max_ of 2.12%.^[^ ^74^ ^]^

#### 3.2.2 Surface Passivation

The poor film quality of perovskites inevitably hinders the realization of efficient and stable blue PeLEDs. The rapid crystallization of the Cl/Br mixed halide perovskites usually induces inhomogeneous and inconsecutive film morphology, leading to high leakage current and poor luminescence. Particularly, quasi‐2D perovskites possess some disadvantages such as low‐order phases and defect‐induced nonradiative recombination, which severely restrict the luminescence efficiency and stability. By introducing 2D PEA_2_PbBr_4_ into quasi‐2D PA_2_(CsPbBr_3_)_*n*−1_PbBr_4_ solution, Ren et al. fabricated a profitable blue‐emission quasi‐2D perovskite of PEA_2−*x*_PA_2_(CsPbBr_3_)_*n*−1_PbBr_4_ with the low‐*n* phase suppression and the efficient ET.^[^ ^75^ ^]^ As a result, a superfluous amount of PEABr could efficaciously passivate the quasi‐2D perovskite, and the blue PeLED (*λ*_EL_ = 488 nm) showed an EQE_max_ of 7.51%, a *L*_max_ of 1765 cd m^−2^, and the improved *T*_50_ of 66 min. Meanwhile, Dong et al. presented a different approach that utilized a solution‐based ligand exchange to passivate the film of close‐packed perovskite NCs with a bipolar shell, consisting of an interior anion shell, an external cation shell and solvents of polar molecules (Figure 4f).^[^ ^76^ ^]^ This strategy could simultaneously control the colloidal stability and short‐ligand passivation, whereas the quantum confinement was retained in bipolar‐shell‐stabilized CsPbBr_3_ perovskite NCs (with a diameter of 4 nm) (Figure 4g). An excellent blue PeLEDs based on perovskite NCs were fabricated with a near‐unity PLQY (surpassing 90%) (Figure 4h) and an EQE_max_ of 12.3% (Figure 4i). In addition, the *T*_50_ reached 20 min at an initial luminance of 90 cd m^−2^, demonstrating that the bipolar‐shell perovskite NCs could improve the device stability.

### 3.3 Imbalanced Charge Injection

In addition to the abundant nonradiative recombination centers in the perovskite films, the mismatched energy‐level alignment between perovskites and the adjacent charge‐transport layers will cause the charge accumulation at the perovskite interfaces, giving rise to the enhancement of Auger nonradiative recombination and the accelerated decomposition of the perovskite films.^[^ ^77^ ^]^ Correspondingly, the operational lifetime and spectral stability of blue PeLEDs will be decreased significantly. This situation is more serious for blue PeLEDs than that of green and red counterparts, as blue‐emission perovskites possess the deeper valence band maximum (VBM) and higher conduction band minimum (CBM). For example, the VBM of blue‐emission perovskites are commonly deeper than the highest occupied molecular orbital (HOMO) of the typical hole‐transport layer (HTL), leading to a large hole‐injection barrier as compared with the injection barrier for electrons.^[^ ^78^ ^]^ Furthermore, the different carrier mobilities of electron‐transport layer (ETL) and HTL cause the discrepant charge‐transport rate and thus an imbalanced charge injection.^[^ ^79^ ^]^ The electron leakage toward HTL will cause irreversible physical damage to HTL.^[^ ^80^, ^81^, ^82^, ^83^ ^]^ To balance the injection of electrons and holes, it is highly desirable to optimize the device architecture of blue PeLEDs by adopting the suitable ETL and HTLs with well‐matched energy‐level alignment and charge mobilities.

**Figure 5:**
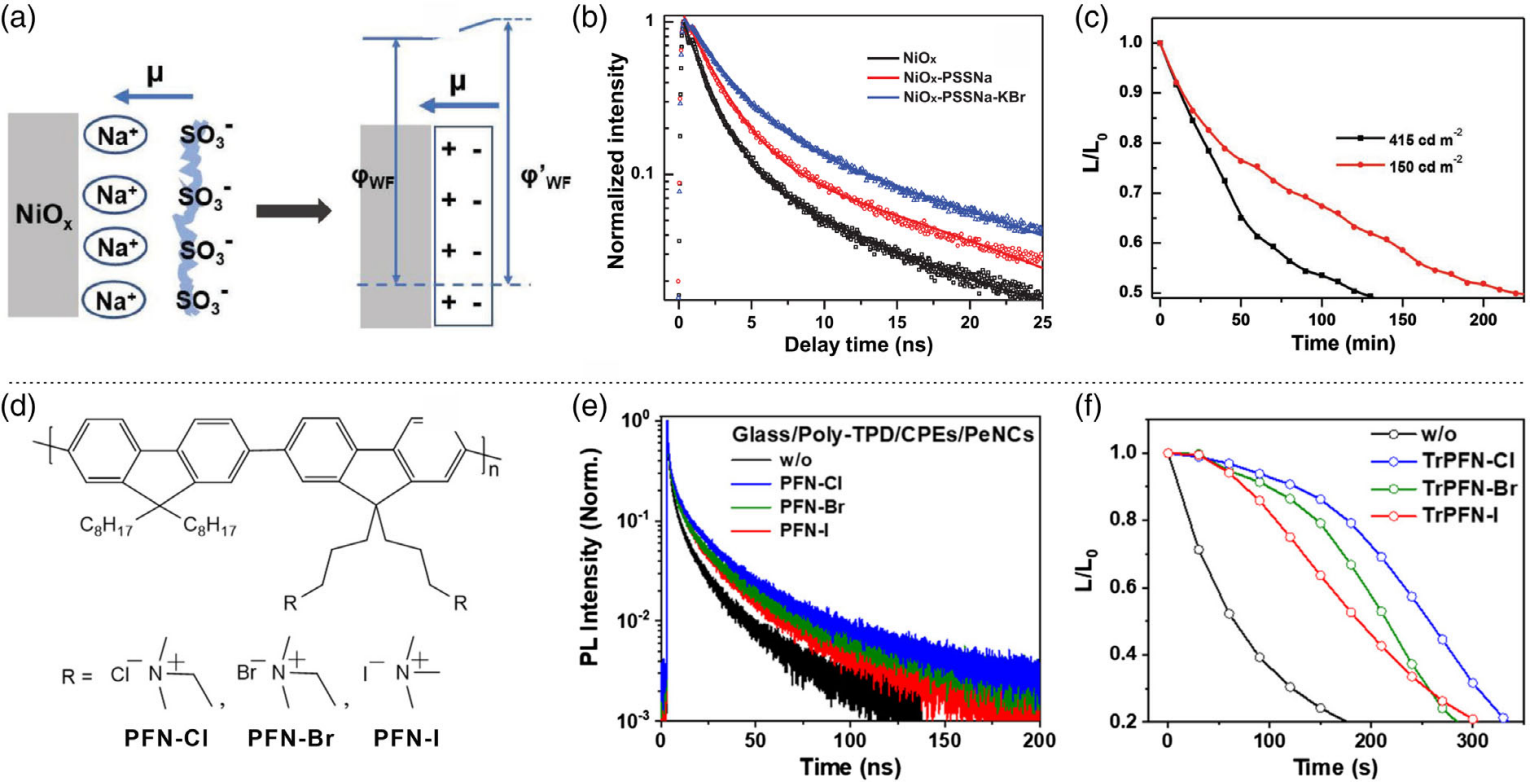
Balanced charge injection via interfacial engineering. a) Schematic of dipole moment formation on NiO_*x*_ by PSSNa and the increased work function of NiO_*x*_‐PSSNa film. b) TRPL decay profiles of perovskites on NiO_*x*_ and NiO_*x*_‐PSSNa, and perovskite with KBr additive on NiO_*x*_‐PSSNa. c) Luminance decay of blue PeLEDs at a constant current. a–c) Reproduced with permission.^[^ ^77^ ^]^ Copyright 2019, Wiley‐VCH. d) Molecular structure of PFN‐Cl, PFN‐Br, and PFN‐I. e) TRPL decay profiles of perovskite NCs on different glass/poly‐TPD with PFN‐Cl, PFN‐Br, and PFN‐I. f) Luminance decay of blue devices with different interface modifications. d–f) Reproduced with permission.^[^ ^84^ ^]^ Copyright 2020, American Chemical Society.

To balance the charge injection and suppress the nonradiative decays, Ren et al. designed a bi‐layer HTL structure using the dipole characteristics of PSSNa on NiO_*x*_ to improve hole transport.^[^ ^77^ ^]^ In this way, the hole‐injection barrier was minimized (**Figure** **5**a), and the nonradiative recombination at the interface between NiO_*x*_ and quasi‐2D perovskite film was significantly suppressed (Figure 5b), yielding a sky‐blue PeLED (*λ*_EL_ = 492 nm) with an EQE_max_ of 1.45% and a *L*_max_ of 4359 cd m^−2^. More importantly, the *T*_50_ operational lifetimes were enhanced to 220 and 120 min, at the initial luminance of 150 and 415 cd m^−2^ (Figure 5c), respectively. Zheng et al. used the solution‐processed poly(9,9‐dioctyl‐fluorene‐*co*‐*N*‐(4butylphenyl)diphenylamine) (TFB)/Li‐doped poly(vinylcarbazole) (PVK) bilayered HTL in blue‐emission quantum‐dot LEDs, and the bilayer HTL provided a stepwise energy‐level structure for not only reducing the hole‐injection barrier but also facilitating the hole transport.^[^ ^80^ ^]^ The TFB/Li‐doped PVK‐based quantum‐dot LEDs record a *L*_max_ of 5829 cd m^−2^ and an EQE_max_ of 5.37%. Shin et al. demonstrated an interfacial engineering strategy with conjugated polyelectrolytes PFN‐X (X = Cl, Br, or I) (Figure 5d), functionalized PFN (poly[(9,9‐bis(3′‐(*N*,*N*‐dimethylamino)propyl)‐2,7‐fluorene)‐*alt*‐2,7‐(9,9‐dioctylfluorene)]) with halide anions, between HTL and perovskite NCs.^[^ ^84^ ^]^ The PFN‐X exhibited the effective electron blocking capability, leading to the well‐balanced charge recombination with the reduced hole‐injection barrier through a high interfacial dipole moment (Figure 5e). Thus, the blue PeLEDs (*λ*_EL_ = 470 nm) show an EQE_max_ of 1.34% with enhanced spectral operating stability (Figure 5f).

Due to the poor charge‐transport properties of quasi‐2D perovskites, an enormous efficiency gap exists in PL and EL. Liu et al. used an emissive layer containing quantum‐confined perovskite NCs embedded in quasi‐2D perovskite, achieving a blue PeLED (*λ*_EL_ = 483 nm) with an EQE_max_ of 9.5% and *T*_50_ of 250 min.^[^ ^79^ ^]^ It has also been observed that incomplete internal ET may lead to multiple EL peaks due to spatial and dielectric confinements in quasi‐2D perovskite. Therefore, Wang et al. presented a deep‐blue PeLED (*λ*_EL_ = 445 nm) based on Cs_3_Cu_2_I_5_ perovskite NCs with a high PLQY of 87% and an EQE_max_ of ≈1.12%.^[^ ^78^ ^]^ More importantly, this device exhibited the unchanged EL peak position regardless of the applied bias, indicating a novel way to improve the luminescence stability of the deep‐blue PeLEDs.^[^ ^85^ ^]^

### 3.4 Optical Loss

For a LED built in a standard substrate emitting architecture, the EQE is determined by the internal quantum efficiency and light outcoupling efficiency (*η*_out_) according to the equation EQE = IQE × *η*_out_, in which IQE is the internal quantum efficiency. Upon the utilization of composition engineering, dimensional modulation, and surface passivation, the PLQY of perovskite materials has been dramatically boosted and the IQE of PeLEDs is approaching 100%. However, the EQE is severely limited by the low *η*_out_ that is ≈20%. The majority of internally generated light is confined in the waveguide mode due to largely different refractive indices between perovskite and glass substrate as well as in the substrate mode due to total internal reflection (TIR) at the glass/air interface.^[^ ^82^, ^83^, ^86^ ^]^ In addition to the waveguide and substrate modes, the optical loss induced by surface plasmon mode should be considered, which originates from the longitudinal oscillation of electrons between metal and dielectronic layers.^[^ ^87^ ^]^ Meng et al. demonstrated that the device structure consisting of transparent electrode/high‐index transport layer/perovskite emitter/low‐index transport layer/reflective electrode could raise *η*_out_ to 12.0%, 17.5%, and 31.2% for blue, green, and red PeLEDs by reducing the optical loss in surface plasmon and waveguide mode.^[^ ^86^ ^]^ Therefore, the low *η*_out_ leaves considerable opportunity for a substantial boost in EQE of blue PeLEDs if the optical loss can be efficiently suppressed, including waveguide mode,^[^ ^86^, ^88^, ^89^, ^90^, ^91^, ^92^, ^93^, ^94^, ^95^, ^96^, ^97^ ^]^ surface plasmon mode,^[^ ^98^, ^99^, ^100^, ^101^, ^102^, ^103^, ^104^, ^105^ ^]^ and substrate mode.^[^ ^101^ ^]^

**Figure 6:**
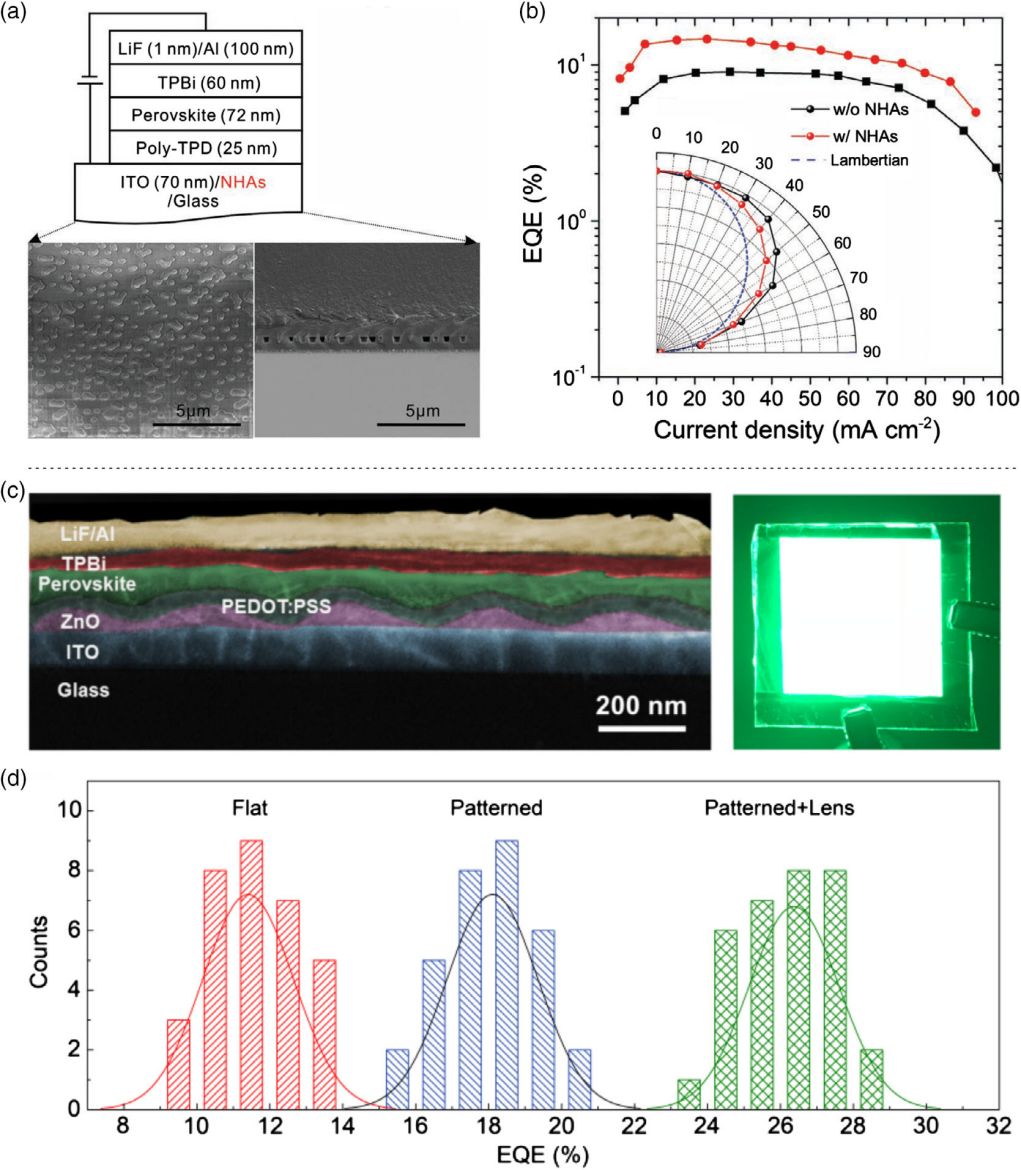
Light outcoupling enhancement in PeLEDs. a) Device structure and scanning electron microscopy (SEM) images of PeLEDs with NHAs. b) EQE versus current density. Inset shows the angle‐dependent emission intensity distributions compared with an ideal Lambertian pattern. a,b) Reproduced with permission.^[^ ^106^ ^]^ Copyright 2019, Wiley‐VCH. c) Cross‐sectional SEM image of the patterned PeLED and a photo of the device with a size of 3.5 × 3.5 cm^2^ in operation. d) Histogram of EQEs of various devices. c,d) Reproduced with permission.^[^ ^99^ ^]^ Copyright 2019, Wiley‐VCH.

Many techniques have been implemented for the light outcoupling enhancement for various LED technologies, including the use of photonic structure at the appropriate device interfaces, refractive‐index coupling layers, microlens arrays, diffraction gratings, and so on.^[^ ^86^, ^88^, ^89^, ^90^, ^91^, ^92^, ^93^, ^94^, ^95^, ^96^, ^97^, ^98^, ^99^, ^100^, ^101^, ^102^, ^103^, ^104^, ^105^, ^106^, ^107^ ^]^ Jeon et al. used a randomly distributed nanohole array (NHA) embedded in a SiN_*x*_ layer between ITO and glass substrate to facilitate the outcoupling of the waveguide mode (**Figure** **6**a).^[^ ^106^ ^]^ High‐efficiency red/near‐infrared PeLEDs with an EQE_max_ of 14.6% were realized (Figure 6b). Shen et al. presented a moth‐eye nanostructures (MEN)‐based outcoupling structure at the front electrode/perovskite interface by inserting a nanoimprinted ZnO layer to release the light trapped in the waveguide mode (Figure 6c).^[^ ^99^ ^]^ A half‐ball lens was further used to outcouple the light trapped in the substrate mode, resulting in an extremely efficient green‐emission CsPbBr_3_ PeLEDs with an EQE_max_ of 28.2% (Figure 6d). Cao et al. proposed the light extraction from the waveguide mode in all directions by preventing the leakage current with thin organic insulating layer between perovskite submicrometric platelets, resulting in an EQE_max_ of 20.7% for infrared PeLEDs.^[^ ^107^ ^]^

### 3.5 Toxicity

Although blue PeLEDs possess broad prospects, their application is still limited by two aspects: chemical/thermal instability and toxicity. The chemical/thermal instability will affect the operational lifetime of the devices, whereas the use of Pb ions in perovskites may have the potential influence on health and the environmental pollution. The ecofriendly Pb‐free halide perovskites have drawn considerable attention as a promising alternative for the practical applications. Similar to Pb^2+^, Sn^2+^ has a *n*s^2^ outermost electronic configuration, and the antibonding hybridization with halide anion is favorable to the formation of shallow defect states for CT and carrier extraction.^[^ ^108^, ^109^, ^110^, ^111^ ^]^ In addition, the Pb^2+^ ions in the 3D perovskite framework of corner‐sharing octahedral mode can be replaced by cation pair of monovalent metals (such as Na^+^, Ag^+^) and trivalent metals (such as Sb^3+^, Bi^3+^) based on the principle of cation transformation.

**Figure 7:**
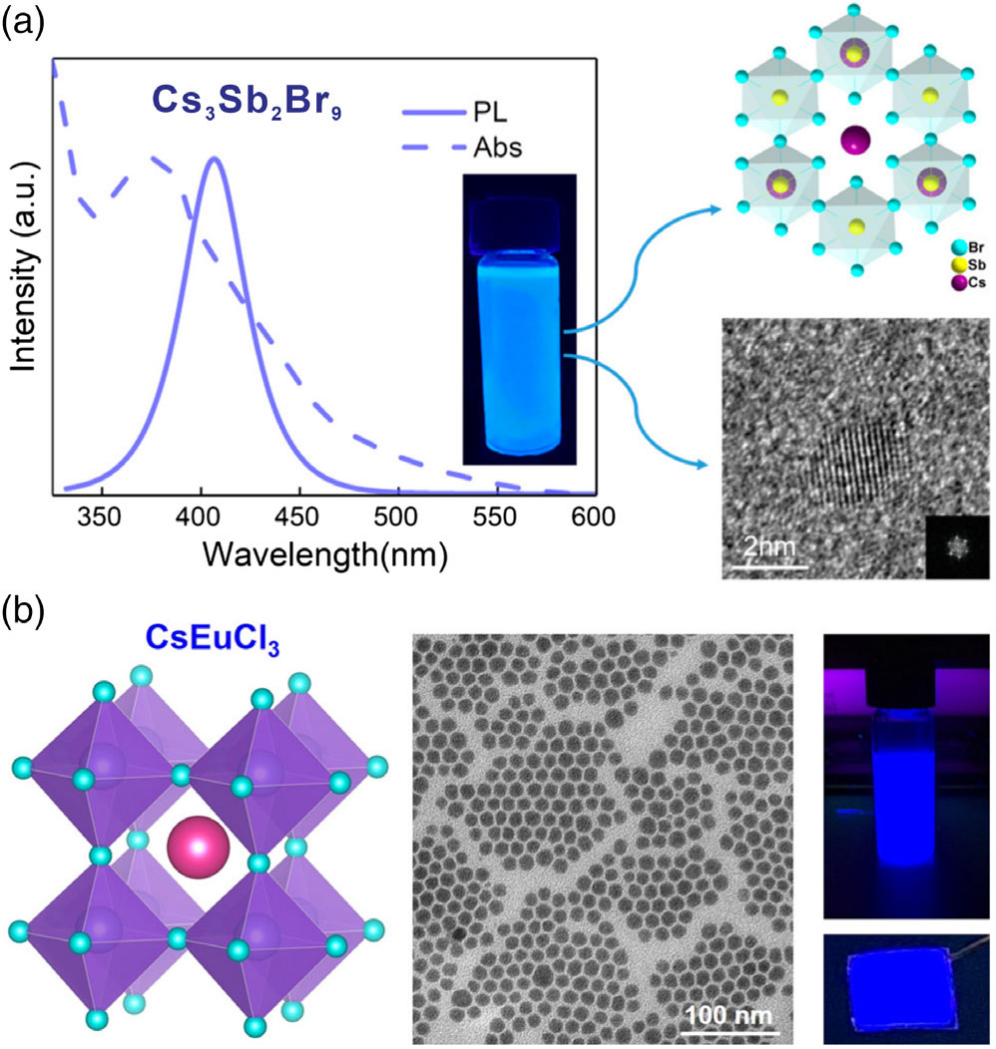
Lead‐free inorganic halide perovskite NCs. a) Heterovalent substitution of divalent lead (Pb^2+^) with trivalent antimony (Sb^3+^) for 2D‐layered Cs_3_Sb_2_Br_9_ inorganic perovskite NCs with an emission at 410 nm. Reproduced with permission.^[^ ^113^ ^]^ Copyright 2017, American Chemical Society. b) The synthesis of CsEuCl_3_ NCs with a diameter of ≈15 nm and an emission at 435 nm. Reproduced with permission.^[^ ^114^ ^]^ Copyright 2020, American Chemical Society.

In 2016, Leng et al. fabricated blue‐emission Pb‐free perovskite NCs of MA_3_Bi_2_X_9_ (X = Cl, Br, I) through a collaborative LARP method, and the PL peaks could be tuned from 360 to 540 nm by controlling the anion composition ratios.^[^ ^112^ ^]^ The deep‐blue PeLEDs were achieved with an EL peak at 430 nm and the PLQY of 12%. Subsequently, Sb‐based perovskite has been proved to possess higher absorption coefficient than that of Bi‐based counterparts. Zhang et al. successfully fabricated Cs_3_Sb_2_Br_9_ perovskite NCs with an emission at 410 nm (**Figure** **7**a), which reached a PLQY of 46% and FWHM of 41 nm (vs a FWHM of MA_3_Bi_2_Br_9_ is 62 nm).^[^ ^113^ ^]^ Liu et al. prepared Cs_2_AgInCl_6_ and Bi‐doped Cs_2_AgInCl_6_ NCs by the thermal injection method with oleic acid, oleylamine, and octadecene as ligands and solvents.^[^ ^108^ ^]^ Undoped Cs_2_AgInCl_6_ NCs showed blue emission at 470 nm, whereas Bi‐doped Cs_2_AgInCl_6_ NCs showed wide orange emission around 580 nm with the greatly improved PLQY up to 11.4%. Recently, Huang et al. developed a colloidal solution‐phase synthetic approach to obtain homogeneous size‐distribution single‐crystalline CsEuCl_3_ NCs, which exhibited a PLQY of 2% at 435 nm with a narrow FWHM of 19 nm (Figure 7b).^[^ ^114^ ^]^ In addition to the improvement in color purity and the degree of saturation, the embedment of CsEuCl_3_ NCs into a polymer matrix could increase the device stability under continuous laser irradiation. Chen et al. reported sky‐blue Cu‐treated halide perovskite NCs by adding CuCl_2_ to Cs_*x*_FA_1−*x*_PbBr_3_ NCs, exhibiting an EQE_max_ of 5.02%.^[^ ^115^ ^]^

## 4 Conclusion

This Review has systematically summarized the factors that restrict the EL and stability of blue PeLEDs and the recent progress to improve luminescence efficiency and stability. The methods to optimize blue‐emission perovskite materials, enhance luminescence stability, balance charge injection, and minimize the optical loss are discussed.

First, the fabrication of homogeneous perovskite films is of utmost importance to achieve high‐performance blue PeLEDs with high luminance and robust stability. Several methods are usually utilized to manipulate rough film morphology and extensive pinholes by controlling the perovskite crystallization rate, optimizing the deposition method, and mixing the polymeric additives into perovskite precursors. In addition, it is a crucial challenge to improve the stability of perovskite NC films, as the degradation of perovskite NC films can lead to a deterioration of the optical properties, such as low PLQY, reduced device efficiency, and spectral variation. In addition, the phase transition of all‐inorganic perovskite NCs can take place between three photoactive black phases (e.g., α phase [cubic], β phase [tetragonal], γ phase [orthorhombic]), and the photo‐inactive yellow δ phase (orthorhombic). To address this problem, replacing Pb^2+^ with B‐site dopants such as Mn^2+^ can provide an effective method to enhance the stability in blue‐emission perovskite NCs.

Second, the precise regulation of the emission wavelength is necessary for the fabrication of blue PeLEDs. Two methods are widely used to obtain the blue emission. One is the composition engineering by controlling the halogen components to tune the bandgap of mixed halide perovskite. Unfortunately, the phase segregation will occur in the mixed halide perovskites due to the electric field‐induced migration of halogen ions, causing an undesirable wavelength shift from blue to green. The other is the dimensional engineering for quasi‐2D perovskite by modulating the low‐dimensional domain with the controlled layer number of inorganic octahedrons. However, this approach would attenuate the color stability because of multiple emission peaks that arise from mixed phases.

Third, the mismatched energy‐level alignment in PeLEDs is detrimental to the device performance. Interlayers or interfacial solutions provide the effective pathways to facilitate charge injection into the perovskite emitters. The selection of the suitable ETL and HTL with well‐matched charge‐transport capabilities are commonly used to balance charge injection and suppress the nonradiative recombination due to the interfacial charge accumulation. Although 2′,2′(1,3,5‐benzoic acid triyl)‐tris(1‐phenyl‐1‐H‐benzimidazole (TPBi) is widely used as a ETL, its carrier mobility is one order of magnitude higher than that of the widely used HTLs, leading to the imbalanced charge injection between electrons and holes. In addition, the incorporation of inorganic charge‐transport materials holds the great potential to improve the operational stability because of their high moisture resistance and oxygen resistance.

Fourth, there is huge room for improvement of *η*_out_. To unlock the full potential of blue PeLEDs, minimizing the optical loss is required without distorted output spectra and limited enhancement at special viewing angles. However, the light outcoupling enhancement is rarely reported for blue PeLEDs. Many techniques have been investigated to enhance *η*_out_ for other LED technologies with the substrate surface modifications and the integration of nanostructures inside the devices for the waveguided light outcoupling. It is thus reasonable to imitate these design guidelines in blue PeLEDs as a potential direction to improve device performance and stability.

Finally, the intrinsic toxicity of Pb‐based perovskite materials is of great concern for blue PeLEDs. Replacing Pb^2+^ cations with the less toxic group IV metal ion is a simple method to fabricate Pb‐free perovskites. Meanwhile, the double perovskites with a general formula of A_2_M^+^M^3+^X_6_ have emerged as a promising alternative to Pb‐based halide perovskites by replacing two toxic Pb^2+^ ions with one M^+^ cation and one M^3+^ cation. Recently, rare‐earth‐metal halide perovskites are regarded as potential perovskite candidates on account of their superior luminance properties. For example, the bulk crystal of hybrid europium halide perovskite consisting of (C_4_H_9_NH_3_)_2_EuI_4_ and CsEuBr_3_ processes strong blue emission with a narrow FWHM of 25–35 nm. However, it is still difficult to control the uniformity and morphology of europium halide perovskite, which is synthesized by solid‐state methods. Therefore, research directions should pay more attention to improve the synthetic route of europium or other rare‐earth halide perovskites in the future to form perovskite films of uniformity and homogeneity. Moreover, the solubility of rare‐earth precursors remains a challenge for the fabrication of blue PeLEDs.

Therefore, as a crucial component of display techniques, blue PeLEDs are still suffering from poor device performance and severe instability both in long‐term operation and emission spectrum. More efforts are still needed to continuously improve the EL efficiency and stability toward the practical applications of blue PeLEDs.

## Conflict of Interest

The authors declare no conflict of interest.

## References

[1] Y. Fu , Q. Zhang , D. Zhang , Y. Tang , L. Shu , Y. Zhu , Z. Fan , Adv. Funct. Mater. 2020, 30, 2002913.

[2] J. Guo , T. Liu , M. Li , C. Liang , K. Wang , G. Hong , Y. Tang , G. Long , S. Yu , T. Lee , W. Huang , G. Xing , Nat. Commun. 2020, 11, 3361. 32681066 10.1038/s41467-020-17096-6 PMC7368017

[3] H. Kanda , N. Shibayama , A. J. Huckaba , Y. Lee , S. Paek , N. Klipfel , C. Roldán-Carmona , V. I. E. Queloz , G. Grancini , Y. Zhang , M. Abuhelaiqa , K. T. Cho , M. Li , M. D. Mensi , S. Kinge , M. K. Nazeeruddin , Energy Environ. Sci. 2020, 13, 1222.

[4] J. Yao , L. Wang , K. Wang , Y. Yin , H. Yao , Sci. Bull. 2020, 65, 1150. 10.1016/j.scib.2020.03.036 36659143

[5] T. Miyasaka , A. Kulkarni , G. M. Kim , S. Öz , A. K Jena , Adv. Energy Mater. 2020, 10, 1902500.

[6] P. You , G. Li , G. Tang , J. Cao , F. Yan , Energy Environ. Sci. 2020, 13, 1187.

[7] Q. Shang , M. Li , L. Zhao , D. Chen , S. Zhang , S. Chen , P. Gao , C. Shen , J. Xing , G. Xing , B. Shen , X. Liu , Q. Zhang , Nano Lett. 2020, 20, 6636. 32786951 10.1021/acs.nanolett.0c02462

[8] J. N. Yang , Y. Song , J. S. Yao , K. H. Wang , J. J. Wang , B. S. Zhu , M. M. Yao , S. U. Rahman , Y. F. Lan , F. J. Fan , H. B. Yao , J. Am. Chem. Soc. 2020, 142, 2956. 31902206 10.1021/jacs.9b11719

[9] J. Miao , F. Zhang , J. Mater. Chem. C 2019, 7, 1741.

[10] F. Zhang , K. Zhu , Adv. Energy Mater. 2020, 10, 1902579.

[11] P. Vashishtha , M. Ng , S. B. Shivarudraiah , J. E. Halpert , Chem. Mater. 2018, 31, 83.

[12] J. Song , J. Li , X. Li , L. Xu , Y. Dong , H. Zeng , Adv. Mater. 2015, 27, 7162. 26444873 10.1002/adma.201502567

[13] A. Sadhanala , S. Ahmad , B. Zhao , N. Giesbrecht , P. M. Pearce , F. Deschler , R. L. Hoye , K. C. Gödel , T. Bein , P. Docampo , S. E. Dutton , M. F. L. De Volder , R. H. Friend , Nano Lett. 2015, 15, 6095. 26236949 10.1021/acs.nanolett.5b02369 PMC4762541

[14] H.-S. Kim , C.-R. Lee , J.-H. Im , K.-B. Lee , T. Moehl , A. Marchioro , S.-J. Moon , R. Humphry-Baker , J.-H. Yum , J. E. Moser , M. Grätzel , N.-G. Park , Sci. Rep. 2012, 2, 591. 22912919 10.1038/srep00591 PMC3423636

[15] D. Shi , V. Adinolfi , R. Comin , M. Yuan , E. Alarousu , A. Buin , Y. Chen , S. Hoogland , A. Rothenberger , K. J. S. Katsiev , Science 2015, 347, 519. 25635092 10.1126/science.aaa2725

[16] N. K. Kumawat , X. K. Liu , D. Kabra , F. Gao , Nanoscale 2019, 11, 2109. 30663760 10.1039/c8nr09885a PMC6369678

[17] J. Cha , H. Lee , S. H. Kim , K. C. Ko , B. J. Suh , O. H. Han , D. Jung , ACS Energy Lett. 2020, 5, 2208.

[18] Q. Zhang , D. Zhang , L. Gu , K. Tsui , S. Poddar , Y. Fu , L. Shu , Z. Fan , ACS Nano 2020, 14, 1577. 31944666 10.1021/acsnano.9b06663

[19] Q. Wang , X. Wang , Z. Yang , N. Zhou , Y. Deng , J. Zhao , X. Xiao , P. Rudd , A. Moran , Y. Yan , J. Huang , Nat. Commun. 2019, 10, 5633. 31822670 10.1038/s41467-019-13580-w PMC6904584

[20] H. Kim , J. S. Kim , J. Heo , M. Pei , I. Park , Z. Liu , H. J. Yun , M. Park , S. Jeong , Y. Kim , J. Park , E. Oveisi , S. Nagane , A. Aadhanala , L. Zhang , J. Kweon , S. K. Lee , H. Yang , H. M. Jang , R. H. Friend , K. P. Loh , M. K. Nazeeruddin , N. Park , T. Lee , Nat. Commun. 2020, 11, 3378. 32632144 10.1038/s41467-020-17072-0 PMC7338442

[21] Q. Li , Y. Yang , W. Que , T. Lian , Nano Lett. 2019, 19, 5620. 31244208 10.1021/acs.nanolett.9b02145

[22] T. Cheng , C. Qin , S. Watanabe , T. Matsushima , C. Adachi , Adv. Funct. Mater. 2020, 30, 2001816.

[23] Y. Jin , Z. K. Wang , S. Yuan , Q. Wang , C. Qin , K. L. Wang , C. Dong , M. Li , Y. Liu , L. Liao , Adv. Funct. Mater. 2020, 30, 1908339.

[24] Y. J. N. P. Nanishi , Nat. Photonics 2014, 8, 884.

[25] M. Auf der Maur , A. Pecchia , G. Penazzi , W. Rodrigues , A. Di Carlo , Phys. Rev. Lett. 2016, 116, 027401. 26824564 10.1103/PhysRevLett.116.027401

[26] F. Kish , F. Steranka , D. DeFevere , D. Vanderwater , K. Park , C. Kuo , T. Osentowski , M. Peanasky , J. Yu , R. M. Fletcher , D. A. Steigerwald , M. G. Craford , V. M. Robbins , Appl. Phys. Lett. 1994, 64, 2839.

[27] K. D. Jandt , R. W. Mills , Dent. Mater. 2013, 29, 605. 23507002 10.1016/j.dental.2013.02.003

[28] M. Shibata , Y. Sakai , D. Yokoyama , J. Mater. Chem. C 2015, 3, 11178.

[29] Z. K. Tan , R. S. Moghaddam , M. L. Lai , P. Docampo , R. Higler , F. Deschler , M. Price , A. Sadhanala , L. M. Pazos , D. Credgington , F. Hanusch , T. Bein , H. J. Snaith , R. H. Friend , Nat. Nanotechnol. 2014, 9, 687. 25086602 10.1038/nnano.2014.149

[30] A. B. Wong , Y. Bekenstein , J. Kang , C. S. Kley , D. Kim , N. A. Gibson , D. Zhang , Y. Yu , S. R. Leone , L. Wang , A. P. Alivisatos , P. Yang , Nano Lett. 2018, 18, 2060. 29504759 10.1021/acs.nanolett.8b00077

[31] T. Chiba , Y. Hayashi , H. Ebe , K. Hoshi , J. Sato , S. Sato , Y. Pu , S. Ohisa , J. J. N. P. Kido , Nat. Photonics 2018, 12, 681.

[32] Y. Ke , N. Wang , D. Kong , Y. Cao , Y. He , L. Zhu , Y. Wang , C. Xue , Q. Peng , F. Gao , W. Huang , J. Wang , J. Phys. Chem. Lett. 2018, 10, 380. 10.1021/acs.jpclett.8b03664 30596240

[33] Q. Wang , J. Ren , X. F. Peng , X. X. Ji , X. H. Yang , ACS Appl. Mater. Interfaces 2017, 9, 29901. 28812341 10.1021/acsami.7b07458

[34] K. Wang , Y. Peng , J. Ge , S. Jiang , B. Zhu , J. Yao , Y. Yin , J. Yang , Q. Zhang , H. J. A. P. Yao , ACS Photonics 2018, 6, 667.

[35] J. Xing , Y. Zhao , M. Askerka , L. N. Quan , X. Gong , W. Zhao , J. Zhao , H. Tan , G. Long , L. Gao , Z. Yang , O. Voznyy , J. Tang , Z. H. Lu , Q. Xiong , E. H. Sargent , Nat. Commun. 2018, 9, 3541. 30166537 10.1038/s41467-018-05909-8 PMC6117319

[36] M. Lu , Y. Zhang , S. Wang , J. Guo , W. W. Yu , A. L. Rogach , Adv. Funct. Mater. 2019, 29, 1902008.

[37] X. Zhao , Z. Tan , Nat. Photonics 2019, 14, 215.

[38] H. P. Kim , J. Kim , B. S. Kim , H. M. Kim , J. Kim , A. R. B. M. Yusoff , J. Jang , M. K. Nazeeruddin , Adv. Opt. Mater. 2017, 5, 1600920.

[39] F. Yuan , C. Ran , L. Zhang , H. Dong , B. Jiao , X. Hou , J. Li , Z. Wu , ACS Energy Lett. 2020, 5, 1062.

[40] P. Du , J. Li , L. Wang , J. Liu , S. Li , N. Liu , Y. Li , M. Zhang , L. Gao , Y. Ma , J. Tang , ACS Appl. Mater. Interfaces 2019, 11, 47083. 31736305 10.1021/acsami.9b17164

[41] M. Yuan , L. N. Quan , R. Comin , G. Walters , R. Sabatini , O. Voznyy , S. Hoogland , Y. Zhao , E. M. Beauregard , P. Kanjanaboos , Z. Lu , D. H. Kim , E. H. Sargent , Nat. Nanotechnol. 2016, 11, 872. 27347835 10.1038/nnano.2016.110

[42] H. Hu , T. Salim , B. Chen , Y. M. Lam , Sci. Rep. 2016, 6, 33546. 27633084 10.1038/srep33546 PMC5025709

[43] Q. Shang , Y. Wang , Y. Zhong , Y. Mi , L. Qin , Y. Zhao , X. Qiu , X. Liu , Q. Zhang , J. Phys. Chem. Lett. 2017, 8, 4431. 28845670 10.1021/acs.jpclett.7b01857

[44] P. Chen , Y. Meng , M. Ahmadi , Q. Peng , C. Gao , L. Xu , M. Shao , Z. Xiong , B. Hu , Nano Energy 2018, 50, 615.

[45] P. Pang , G. Jin , C. Liang , B. Wang , W. Xiang , D. Zhang , J. Xu , W. Hong , Z. Xiao , L. Wang , G. Xing , J. Chen , D. Ma , ACS Nano 2020, 14, 11420. 32812732 10.1021/acsnano.0c03765

[46] S. Chang , Z. L. Bai , H. Z. Zhong , Adv. Opt. Mater. 2018, 6, 1800380.

[47] L. C. Schmidt , A. Pertegás , S. González-Carrero , O. Malinkiewicz , S. Agouram , G. Minguez Espallargas , H. J. Bolink , R. E. Galian , J. Pérez-Prieto , J. Am. Chem. Soc. 2014, 136, 850. 24387158 10.1021/ja4109209

[48] S. Gonzalez Carrero , L. Francés Soriano , M. González Béjar , S. Agouram , R. E. Galian , J. Pérez-Prieto , Small 2016, 12, 5245. 27555293 10.1002/smll.201600209

[49] Q. A. Akkerman , V. D'Innocenzo , S. Accornero , A. Scarpellini , A. Petrozza , M. Prato , L. Manna , J. Am. Chem. Soc. 2015, 137, 10276. 26214734 10.1021/jacs.5b05602 PMC4543997

[50] G. Nedelcu , L. Protesescu , S. Yakunin , M. I. Bodnarchuk , M. J. Grotevent , M. V. Kovalenko , Nano Lett. 2015, 15, 5635. 26207728 10.1021/acs.nanolett.5b02404 PMC4538456

[51] B. Wang , C. Zhang , W. Zheng , Q. Zhang , Z. Bao , L. Kong , L. Li , Chem. Mater. 2020, 32, 308.

[52] F. Zhang , H. Zhong , C. Chen , X. G. Wu , X. Hu , H. Huang , J. Han , B. Zou , Y. Dong , ACS Nano 2015, 9, 4533. 25824283 10.1021/acsnano.5b01154

[53] J. K. Park , J. H. Heo , B. W. Kim , S. H. Im , J. Ind. Eng. Chem. 2020, 92, 167.

[54] L. Protesescu , S. Yakunin , M. I. Bodnarchuk , F. Krieg , R. Caputo , C. H. Hendon , R. X. Yang , A. Walsh , M. V. Kovalenko , Nano Lett. 2015, 15, 3692. 25633588 10.1021/nl5048779 PMC4462997

[55] F. Ye , H. Zhang , P. Wang , J. Cai , L. Wang , D. Liu , T. Wang . Chem. Mater. 2020, 32, 3211.

[56] H. Liu , Z. Wu , H. Gao , J. Shao , H. Zou , D. Yao , Y. Liu , H. Zhang , B. Yang . ACS Appl. Mater. Interfaces 2017, 9, 42919. 29200265 10.1021/acsami.7b14677

[57] K. Thesika , A. V. Murugan , Inorg. Chem. 2020, 59, 6161. 32286803 10.1021/acs.inorgchem.0c00294

[58] Z. Chu , Y. Zhao , F. Ma , C. X. Zhang , H. Deng , F. Gao , Q. Ye , J. Meng , Z. Yin , X. Zhang , J. You , Nat. Commun. 2020, 11, 4165. 32820166 10.1038/s41467-020-17943-6 PMC7441179

[59] S. Yuan , Z. K. Wang , L. X. Xiao , C. F. Zhang , S. Y. Yang , B. B. Chen , H. T. Ge , Q. S. Tian , Y. Jin , L. S. Liao . Adv. Mater 2019, 31, 1904319. 10.1002/adma.201904319 31532872

[60] Y. Shen , K. C. Shen , Y. Q. Li , M. L. Guo , J. K. Wang , Y. C. Ye , F. M. Xie , H. Ren , X. Gao , F. Song , J. X. Tang . Adv. Funct. Mater 2020, 31, 2006736.

[61] S. Yan , W. Tian , H. Chen , K. Tang , T. Lin , G. Zhong , L. Qiu , X. Pan , W. Wang . Adv. Opt. Mater. 2020, 9, 2001709.

[62] J. H. Kim , K. H. Lee , H. D. Kang , B. Park , J. Y. Hwang , H. S. Jang , Y. R. Do , H. Yang , Nanoscale 2015, 7, 5363. 25721494 10.1039/c5nr00426h

[63] B. W. Park , S. I. J. A. M. Seok , Adv. Mater. 2019, 31, 1805337. 10.1002/adma.201805337 30773706

[64] Y. Lin , B. Chen , Y. Fang , J. Zhao , C. Bao , Z. Yu , Y. Deng , P. N. Rudd , Y. Yan , Y. Yuan , J. Huang , Nat. Commun. 2018, 9, 4981. 30478392 10.1038/s41467-018-07438-w PMC6255755

[65] E. Tenuta , C. Zheng , O. Rubel , Sci. Rep. 2016, 6, 37654. 27883032 10.1038/srep37654 PMC5121628

[66] H. Cho , Y. H. Kim , C. Wolf , H. D. Lee , T. W. Lee , Adv. Mater. 2018, 30, 1704587. 10.1002/adma.201704587 29369426

[67] H. Back , G. Kim , J. Kim , J. Kong , T. K. Kim , H. Kang , H. Kim , J. Lee , S. Lee , K. Lee , Energy Environ. Sci. 2016, 9, 1258.

[68] Q. Khan , A. Subramanian , G. Yu , K. Maaz , D. Li , R. U. R. Sagar , K. Chen , W. Lei , B. Shabbir , Y. J. N. Zhang , Nanoscale 2019, 11, 5021. 30839976 10.1039/c8nr09864f

[69] A. Swarnkar , W. J. Mir , A. Nag , ACS Energy Lett. 2018, 3, 286.

[70] P. Pal , S. Saha , A. Banik , A. Sarkar , K. Biswas , Chem. Eur. J. 2018, 24, 1811. 29293285 10.1002/chem.201705682

[71] D. Solis-Ibarra , I. C. Smith , H. I. Karunadasa , Chem. Sci. 2015, 6, 4054. 29218171 10.1039/c5sc01135c PMC5707501

[72] S. Bhattacharyya , D. Rambabu , T. K. Maji , J. Mater. Chem. A 2019, 7, 21106.

[73] N. Mondal , A. De , A. Samanta , ACS Energy Lett. 2018, 4, 32.

[74] S. Hou , M. K. Gangishetty , Q. Quan , D. N. Congreve , Joule 2018, 2, 2421. 10.1002/adma.201706226 29575250

[75] Z. Ren , L. Li , J. Yu , R. Ma , X. Xiao , R. Chen , K. Wang , X. W. Sun , W. J. Yin , W. C. H. Choy , ACS Energy Lett. 2020, 5, 2569.

[76] Y. Dong , Y. K. Wang , F. Yuan , A. Johnston , Y. Liu , D. Ma , M. J. Choi , B. Chen , M. Chekini , S. W. Baek , L. K. Sagar , J. Fan , Y. Hou , M. Wu , S. Lee , B. Sun , S. Hoogland , R. Quintero Bermudez , H. Ebe , P. Todorovic , F. Dinic , P. Li , H. T. Kung , M. I. Saidaminov , E. Kumacheva , E. Spiecker , L. S. Liao , O. Voznyy , Z. H. Lu , E. H. Sargent , Nat. Nanotechnol. 2020, 15, 668. 32632321 10.1038/s41565-020-0714-5

[77] Z. Ren , X. Xiao , R. Ma , H. Lin , K. Wang , X. W. Sun , W. C. H. Choy , Adv. Funct. Mater. 2019, 29, 1905339.

[78] L. Wang , Z. Shi , Z. Ma , D. Yang , F. Zhang , X. Ji , M. Wang , X. Chen , G. Na , S. J. N. L. Chen , Nano Lett. 2020, 20, 3568. 32243171 10.1021/acs.nanolett.0c00513

[79] Y. Liu , J. Cui , K. Du , H. Tian , Z. He , Q. Zhou , Z. Yang , Y. Deng , D. Chen , X. Zuo , Y. Ren , L. Wang , H. Zhu , B. Zhao , D. Di , J. Wang , R. H. Friend , Y. Jin , Nat. Photonics 2019, 13, 1905339.

[80] L. Zheng , G. Zhai , Y. Zhang , X. Jin , L. Gao , Z. Yun , Y. Miao , H. Wang , Y. Wu , B. J. S. Xu , Microstructures 2020, 140, 106460.

[81] J. H. Chang , P. Park , H. Jung , B. G. Jeong , D. Hahm , G. Nagamine , J. Ko , J. Cho , L. A. Padilha , D. C. J. A. n. Lee , ACS Nano 2018, 12, 10231. 30347988 10.1021/acsnano.8b03386

[82] L. Zhao , K. M. Lee , K. Roh , S. U. Z. Khan , B. P. Rand , Adv. Mater. 2019, 31, 1805836. 10.1002/adma.201805836 30412319

[83] B. Zhao , S. Bai , V. Kim , R. Lamboll , R. Shivanna , F. Auras , J. M. Richter , L. Yang , L. Dai , M. Alsari , X.-J. She , L. Liang , J. Zhang , S. Lilliu , P. Gao , H. J. Snaith , J. Wang , N. C. Greenham , R. H. Friend , D. Di , Nat. Photon. 2018, 12, 783.

[84] Y. S. Shin , Y. J. Yoon , J. Heo , S. Song , J. W. Kim , S. Y. Park , H. W. Cho , G.-H. Kim , J. Y. J. A. a. m. Kim , ACS Appl. Mater. Interfaces 2020, 12, 35740. 32633483 10.1021/acsami.0c09968

[85] O. Malinkiewicz , A. Yella , Y. H. Lee , G. M. Espallargas , M. Graetzel , M. Nazeeruddin , H. J. Bolink , Nat. Photonics 2014, 8, 128.

[86] S. S. Meng , Y. Q. Li , J. X. Tang , Org. Electron. 2018, 61, 351.

[87] S. Wu , Z. Li , M. Li , Q. Diao , Y. Lin , F. Liu , A. K. Y. Jen , Nat. Nanotechnol. 2020, 15, 934. 32958933 10.1038/s41565-020-0765-7

[88] I. L. Braly , D. W. deQuilettes , L. M. Pazos-Outón , S. Burke , M. E. Ziffer , D. S. Ginger , H. W. J. N. P. Hillhouse , Nat. Photonics 2018, 12, 355.

[89] W. Brütting , J. Frischeisen , T. D. Schmidt , B. J. Scholz , C. J. Mayr , Phys. Status Solidi 2013, 210, 44.

[90] Q. Zhang , D. Zhang , Y. Fu , S. Poddar , L. Shu , X. Mo , Z. Fan , Adv. Funct. Mater. 2020, 30, 2002570.

[91] Y. Shen , M.-N. Li , Y. Li , F.-M. Xie , H.-Y. Wu , G.-H. Zhang , L. Chen , S.-T. Lee , J.-X. Tang , ACS Nano 2020, 14, 6107. 32223190 10.1021/acsnano.0c01908

[92] Y. J. Li , Y. Lv , C.-L. Zou , W. Zhang , J. Yao , Y. S. Zhao , J. Am. Chem. Soc. 2016, 138, 2122. 26849536 10.1021/jacs.5b12755

[93] K. Wang , W. Sun , J. Li , Z. Gu , S. Xiao , Q. Song , ACS Photonics 2016, 3, 1125.

[94] P. J. Cegielski , S. Neutzner , C. Porschatis , H. Lerch , J. Bolten , S. Suckow , A. R. S. Kandada , B. Chmielak , A. Petrozza , T. Wahlbrink , A. L. Giesecke , Opt. Express 2017, 25, 13199. 28788855 10.1364/OE.25.013199

[95] H. Zhu , Y. Fu , F. Meng , X. Wu , Z. Gong , Q. Ding , M. V. Gustafsson , M. T. Trinh , S. Jin , X. Y. Zhu , Nat. Mater. 2015, 14, 636. 25849532 10.1038/nmat4271

[96] F. J. Walker , R. A. McKee , H. w. Yen , D. E. Zelmon , Appl. Phys. Let. 1994, 65, 1495.

[97] M. Abdi-Jalebi , Z. Andaji-Garmaroudi , S. Cacovich , C. Stavrakas , B. Philippe , J. M. Richter , M. Alsari , E. P. Booker , E. M. Hutter , A. J. J. N. Pearson , Nature 2018, 555, 497. 29565365 10.1038/nature25989

[98] X. B. Shi , Y. Liu , Z. Yuan , X. K. Liu , Y. Miao , J. Wang , S. Lenk , S. Reineke , F. J. A. O. M. Gao , Adv. Opt. Mater. 2018, 6, 1800667.

[99] Y. Shen , L. P. Cheng , Y. Q. Li , W. Li , J. D. Chen , S. T. Lee , J. X. J. A. M. Tang , Adv. Mater. 2019, 31, 1901517. 10.1002/adma.201901517 31012195

[100] G. V. J. C. r. Hartland , Chem. Rev. 2011, 111, 3858. 21434614 10.1021/cr1002547

[101] L. Gu , K. Wen , Q. Peng , W. Huang , J. J. S. Wang , Small 2020, 16, 2001861; 10.1002/smll.202001861 32573954

[102] Q. Shang , S. Zhang , Z. Liu , J. Chen , P. Yang , C. Li , W. Li , Y. Zhang , Q. Xiong , X. Liu , Q. Zhang , Nano Lett. 2018, 18, 3335. 29722986 10.1021/acs.nanolett.7b04847

[103] C. Li , Z. Liu , Q. Shang , Q. Zhang , Adv. Opt. Mater. 2019, 7, 1900279.

[104] Z. Shi , Y. Li , S. Li , X. Li , D. Wu , T. Xu , Y. Tian , Y. Chen , Y. Zhang , B. Zhang , C. Shan , G. Du , Adv. Funct. Mater. 2018, 28, 1707031.

[105] Y. Zhang , H. Sun , S. Zhang , S. Li , X. Wang , X. Zhang , T. Liu , Z. Guo , Opt. Mater. 2019, 89, 563.

[106] S. Jeon , L. Zhao , Y. J. Jung , J. W. Kim , S. Y. Kim , H. Kang , J. H. Jeong , B. P. Rand , J. H. J. S. Lee , Small 2019, 15, 1900135. 10.1002/smll.201900135 30701678

[107] Y. Cao , N. Wang , H. Tian , J. Guo , Y. Wei , H. Chen , Y. Miao , W. Zou , K. Pan , Y. He , H. Cao , Y. Ke , M. Xu , Y. Wang , M. Yang , K. Du , Z. Fu , D. Kong , D. Dai , Y. Jin , G. Li , H. Li , Q. Peng , J. Wang , W. Huang , Nature 2018, 562, 249. 30305742 10.1038/s41586-018-0576-2

[108] Y. Liu , Y. Jing , J. Zhao , Q. Liu , Z. Xia , Chem. Mater. 2019, 31, 3333.

[109] M. Ozaki , Y. Katsuki , J. Liu , T. Handa , R. Nishikubo , S. Yakumaru , Y. Hashikawa , Y. Murata , T. Saito , Y. Shimakawa , Y. Kanemitsu , A. Saeki , A. Wakamiya , ACS Omega 2017, 2, 7016. 31457283 10.1021/acsomega.7b01292 PMC6644770

[110] W. Gao , C. Chen , C. Ran , H. Zheng , H. Dong , Y. Xia , Y. Chen , W. Huang , Adv. Funct. Mater. 2020, 30, 2000794.

[111] Z. Liu , X. Zhao , A. Zunger , L. Zhang , Adv. Electron. Mater. 2019, 5, 1900234.

[112] M. Leng , Z. Chen , Y. Yang , Z. Li , K. Zeng , K. Li , G. Niu , Y. He , Q. Zhou , J. Tang , Angew. Chem. Int. Engl. 2016, 55, 15012. 10.1002/anie.201608160 27791304

[113] J. Zhang , Y. Yang , H. Deng , U. Farooq , X. Yang , J. Khan , J. Tang , H. Song , ACS Nano 2017, 11, 9294. 28880532 10.1021/acsnano.7b04683

[114] J. Huang , T. Lei , M. Siron , Y. Zhang , S. Yu , F. Seeler , A. Dehestani , L. N. Quan , K. Schierle-Arndt , P. Yang , Nano Lett. 2020, 20, 3734. 32348146 10.1021/acs.nanolett.0c00692

[115] F. Chen , L. Xu , Y. Li , T. Fang , T. Wang , M. Salerno , M. Prato , J. Song , J. Mater. Chem. C 2020, 8, 13445.

